# Active Learning Optimisation of Binary Coded Metasurface Consisting of Wideband Meta-Atoms

**DOI:** 10.3390/s23125546

**Published:** 2023-06-13

**Authors:** Parvathy Chittur Subramanianprasad, Yihan Ma, Achintha Avin Ihalage, Yang Hao

**Affiliations:** School of Electronics Engineering and Computer Science, Queen Mary University of London, Mile End Rd, Bethnal Green, London E1 4NS, UK; p.chittursubramanianprasad@qmul.ac.uk (P.C.S.); yihan.ma@qmul.ac.uk (Y.M.); a.a.ihalage@qmul.ac.uk (A.A.I.)

**Keywords:** active learning, optimisation, metasurface design

## Abstract

The design of a metasurface array consisting of different unit cells with the objective of minimizing its radar cross-section is a popular research topic. Currently, this is achieved by conventional optimisation algorithms such as genetic algorithm (GA) and particle swarm optimisation (PSO). One major concern of such algorithms is the extreme time complexity, which makes them computationally forbidden, particularly at large metasurface array size. Here, we apply a machine learning optimisation technique called active learning to significantly speed up the optimisation process while producing very similar results compared to GA. For a metasurface array of size 10 × 10 at a population size of $10^{6}$, active learning took 65 min to find the optimal design compared to genetic algorithm, which took 13,260 min to return an almost similar optimal result. The active learning optimisation strategy produced an optimal design for a 60 × 60 metasurface array 24× faster than the approximately similar result generated by GA technique. Thus, this study concludes that active learning drastically reduces computational time for optimisation compared to genetic algorithm, particularly for a larger metasurface array. Active learning using an accurately trained surrogate model also contributes to further lowering of the computational time of the optimisation procedure.

## 1. Introduction

Metamaterials are three-dimensional man-made substances that can achieve electromagnetic properties, such as negative index of refraction, which cannot be found in naturally occurring materials [1]. The uniqueness of metamaterials derives from their internal microstructures and nanostructures, rather than the chemical composition found in natural materials. The multiple stacks of material layers in metamaterials lead to significant losses and challenges in nano fabrication. Metasurfaces are the two-dimensional equivalent of metamaterials and have been developed to overcome the obstacles that metamaterials are confronted with. Metasurfaces are planar patterned surfaces composed of sub-wavelength periodic arrays of unit cells capable of manipulating the behaviour of light [2]. The principle of operation of metasurfaces is based on the phenomenon of diffraction. There is a vast area of application for metasurfaces, including absorption [3], holographic imaging [4], and wavefront manipulation [5]. The radar cross-section is the electromagnetic signature of an object. When the radar cross-section is large, the object will be easily detectable by radar. A stealth aircraft designed to have low radar detectability—whether it be from land-based installations, guided weapons, or other vehicles—would require a surface with a low radar cross-section. A low radar cross-section improves a platform’s overall survivability through the improved effectiveness of its radar counter-measures. A metasurface structure can effectively decrease radar cross-section scattering due to its distinctive phase manipulation capabilities [6,7]. Decreasing radar cross-section using metasurface has been a widely discussed topic for the last few years. The two main classes of metasurface optimisation are gradient-based and gradient-free methodologies [8,9,10,11]. Gradient-based methods, such as topology optimisation, rely strongly on the initial design [12]. Gradient-free methods, such as genetic algorithm [13] and particle swarm optimisation [14], have a high computational cost, especially when dealing with large parameter spaces. Single-objective approaches either evolve from an initial guess to a final result through evolutionary methods [15] or depend on exhaustive design parameter sweeps using a brute-force EM solver (e.g., based on the finite element method [16]). While the former highly depends on the initial guess and in most cases converges to a local optimum, the latter requires extensive computation. The above-mentioned single-objective approaches fail when the input-output relation is complex or when the number of desired features for a nanostructure grows and the features are computationally demanding. Although multi-objective methods [17,18] are more computationally efficient, obtaining an optimal solution is not guaranteed. Overall, the current optimisation methods for the design of metasurfaces, especially at larger scales, are computationally intensive operations. Alternatively, the rapid development of machine learning (ML) techniques in recent years has successfully been applied for metasurface design [19,20]. As a data-driven approach, ML algorithms are capable of learning the relationship between a metasurface structure and its corresponding electromagnetic properties. Instead of employing numerical algorithms, the trained ML model can solve the electromagnetic problems in a short period of time.

A multilayer perceptron (MLP) artificial neural network (ANN) is used to predict the power coupling efficiency of photonic couplers and is integrated with evolutionary strategy (ES) for optimizing the photonic coupler design [21]. When MLP is combined with ES, the computational time of the optimisation processes is significantly reduced compared to full-wave solvers. The machine learning technique does not require the operations of an evolutionary algorithm, such as reproduction, mutation, recombination, selection, and survival of the fittest. In the work [22], a three-dimensional finite-difference time-domain (FDTD) method is integrated with a machine learning algorithm to design an efficient compact photonic structure. The generation of datasets is a time-consuming offline process, although it is required only one time. In the work [23], rigorous coupled-wave analysis (RCWA) is used to generate 25,000 training examples to train a deep learning model with input features as the structure composed of two silicon nanobars with scalable characteristics including the spectral position, line width, and amplitude of the transmission, and the target is the transmission spectra of the metasurface from the wavelength 1400 nm to 1600 nm. The optimisation of the frequency-selective surface using periodic elements with a repetition period larger than one wavelength is performed for the first time in the work [24] with the help of genetic algorithm to achieve wideband scattering diffusion. A period of several days has been reported as the timespan for global optimisation computation. The coding metasurface designed in the work [25] using genetic algorithm optimisation reports a 6 dB RCS reduction in the frequency band 6.28–9.16 GHz and 6.33–9.41 GHz, respectively, for transverse electric and transverse magnetic polarised normal incident waves. A probabilistic technique known as simulated annealing is used for approximating the global minimum of the function as given in Equation (1) [26].

(1) $$RCS\;reduction=10\times log[\frac{A_{1}\times e^{jP_{1}}+A_{2}\times e^{jP_{2}}}{2}]$$

where $A_{1}$ and $A_{2}$ are the reflection coefficient magnitudes of two artificial magnetic conductor structures, and $P_{1}$ and $P_{2}$ are their reflection phases. The radar cross-section reduction of the proposed coding metasurface in this work is designed from 7 GHz to 20 GHz, but the radar cross-section reduction achieved from 8.7 GHz to 11.3 GHz is not much. Although incorporating the ML algorithm into the evolutionary algorithm accelerates the optimisation process to some extent, it still requires significant computational resources to prepare datasets for model training, especially when the dimension of the metasurface grows large. In this paper, a novel approach is proposed to accelerate the optimisation procedure by employing the active learning algorithm. It is important to note that, to the best of the author’s knowledge, this is the first time that a machine learning approach has been utilised to design a metasurface array of size 60 × 60 with optimal RCS. Mutual coupling between the unit cells is taken into consideration in this work of metasurface array design. The optimisation objective is accomplished in a reasonable time using the active learning methodology. We have also aimed at evaluating the effects of substituting the CST Microwave Studio simulation step in active learning with a prediction made by the accurately trained machine learning model. The well-fitted modelling results demonstrate that this method can be extended to metasurface array structures of arbitrary scale.

## 2. Methods

The objective of this study is to find the optimal design of a metasurface array consisting of two different unit cells, in such a way that the resultant array has the minimum radar cross-section reduction. Genetic algorithm provides an optimal design and works well when using a smaller population size. When considering a larger metasurface array, the population size to be considered also increases. Genetic algorithm is too computationally expensive when entering the realm of larger population size.

### 2.1. Active Learning

**Figure 1:**
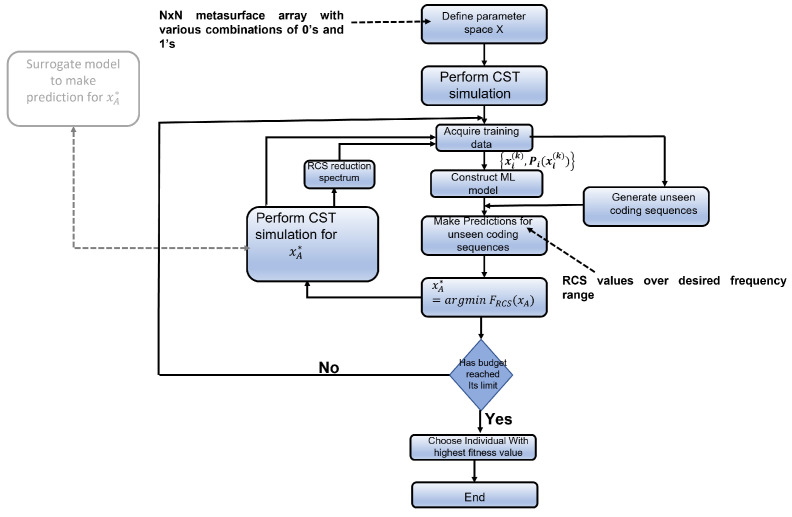
Flowchart of active learning. This figure gives an overview of steps involved in the active learning methodology, which are as follows: generate the dataset to train the machine learning model, query the sample space using the relevant acquisition function, select the suitable data point as per the objective function, perform CST Microwave Studio simulation, add the data with its result to the training set, and retrain the machine learning model. The possibility of substitution of CST simulation with a surrogate trained model is demonstrated by the grey shaded box in the left side of the flowchart.

An active learning procedure was also used to perform the optimisation process as genetic algorithm is a computationally expensive operation. Active learning is a way to select the relevant representative data in any region of the solution space when considering a large solution space for the optimisation. Active learning is very effective in settings where there is a lot of unlabelled data available, but the annotation task is expensive or time-consuming. Active learning is a mature technique, and due to its versatility, it has been applied in a diverse number of settings. The working procedure of active learning, as shown in Figure 1, involves, first, training of a machine learning model using the available labelled dataset, then iteratively retraining the model after querying the subset of unlabelled data points; after finding its target value, the most suitable data point, depending on the problem, is added to the training set. The iteration procedure at first consists of querying the subset of the solution space. The second step is adding the relevant data instances, after suitably labelling them, to the training dataset. The third step is to retrain the model. All of the above three steps can be performed for the required number of times, known as the budget, depending on the available computational resources.

Active learning is a popular algorithm in the material science discipline for the discovery of new materials with desired properties. Active learning is described as an adaptive learning strategy [27,28]. The Bayesian and decision theoretic approach are used in active learning. There are two steps in the process of Bayesian global optimisation, as follows:

- Step 1—A model that can predict the value of the objective function f(x) at unseen data points based on available data $\left(x_{i},y_{i}\right)$ with i=1……,n.
- Step 2—Choosing an acquisition function such that it provides a good decision-making step to direct the future sampling space in the voluminous solution space of the objective function.

The acquisition functions chosen can be any of the following [27]:

- Expected improvement acquisition function—Expected improvement of the objective function f(x) is given by $E\left[I\left(x\right)\right]=E\left[f\left(x\right)-f\left(x^{+}\right)\right]$, and the data point $x^{+}$ for the following iteration is chosen as the one that results in the maximum expected improvement. Since f(x) is normally distributed with mean μ and standard deviation σ, the expected improvement can be written as in Equation (2).
  (2) $$\begin{array}{c} \begin{array}{cc} \; & E\left[I\left(x\right)\right]=\int_{f(x^{+})}^{inf}\left(z-f\left(x^{+}\right)\right)\phi \left(z\right)dz\\ \; & \;=\left(\mu \left(x\right)-f\left(x^{+}\right)\right)\left[\phi \left(\frac{\mu \left(x\right)-f\left(x^{+}\right)}{\sigma (x)}\right)+\sigma \left(x\right)\Phi \left(\frac{\mu \left(x\right)-f\left(x^{+}\right)}{\sigma (x)}\right)\right] \end{array} \end{array}$$
  Note that ϕ and Φ are the standard normal density and cumulative distribution functions.
- Knowledge gradient—In the presence of noise where f(x) values are not exactly known, μ(x) is considered, thus making the new data point $x^{+}$ the one that results in the maximum improvement of μ(x) in the next step (i.e., where the knowledge gradient as shown in Equation (3) is largest).
  (3) $$\begin{array}{c} KG\left(x\right)=\sigma \left[\phi \left(\frac{\mu \left(x\right)-f\left(x^{+}\right)}{\sigma (x)}\right)\right]+\Phi \left(\frac{\mu \left(x\right)-f\left(x^{+}\right)}{\sigma (x)}\right) \end{array}$$
- Mean objective cost of uncertainty—The mean objective cost of uncertainty (MOCU) is given as in Equation (4) for each parameter value θ. The data point $x^{+}$ for the next iteration is chosen based on the one that reduces the MOCU the most.
  (4) $$\begin{array}{c} \begin{array}{c} MOCU=E_{\theta }\left[f_{\theta }\left(x^{+}\right)-f_{\theta }\left(x_{robust}\right)\right]\\ where\;x_{robust}=argmax_{x}E_{\theta }f\left(x\right) \end{array} \end{array}$$
  $x_{robust}$ maximises the expected value of f(x) over the unknown parameters, θ, assuming we have a prior distribution for θ to allow us to evaluate the expected value. The new data point $x^{*}$ is given by Equation (5).
  (5) $$x^{*}=argmin_{x}E_{y|x}\left(E_{\theta |x}\left[f_{\theta |x}\left(x^{+}\right)-f_{\theta |x}\left(x_{robust}\right)\right]\right)$$

In the field of machine learning, active learning dedicated to optimal experiment design has existed in science since the 18th century, when Laplace used it to guide his discovery of celestial mechanics [29]. The trade-off between exploration of the solution space of the optimisation problem and exploitation, which is the final aim to find the optimal result, is the key consideration in active learning. Active learning is applied in the field of material discovery [30]. The drawback of automating medical image interpretation come from the fact that the cost, effort, and time taken to acquire correctly annotated training datasets are significant, in turn resulting in difficulty in training an accurate machine learning model. Application of the active learning strategy for medical image analysis has been discussed in [31], which overcomes the annotation bottleneck and reduces the costs associated with developing deep-learning-enabled systems from unannotated data. In the work of [32], an active learning methodology is applied for the classification of cancer pathology reports. In [33], active learning is used for predicting heart disease. In the work [34], reliability analysis reveals how an active learning framework can be used to enhance accuracy while alleviating the computational cost. Active learning is used as a basis of recommender systems in [35].

The ingenuous approach of exploitation in active learning is where the algorithm always chooses the data point resulting in the maximum or minimum of the objective function, depending on the optimisation problem. In more polished approaches, the algorithm uses policies such as maximum likelihood of improvement, minimum uncertainty, and maximum expected improvement to select the data point. Choosing optimal datapoints using the aforementioned policies requires the machine learning model to return a prediction and also the uncertainty of prediction. In our work, we have used maximum expected improvement to select the data point to be queried and included in the training set.

### 2.2. Developing the Machine Learning Model

#### 2.2.1. Data Generation

**Figure 2:**
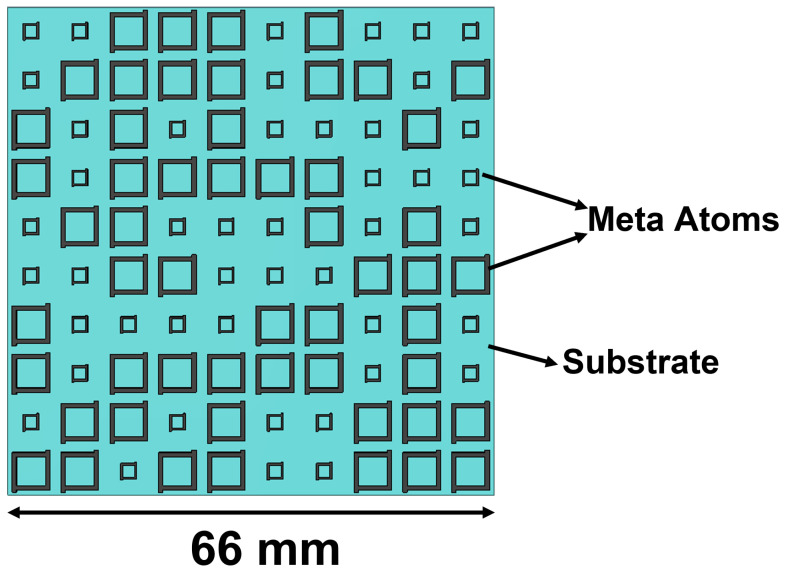
Metasurface array structure: the layout of the metasurface array considered in this study.

**Figure 3:**
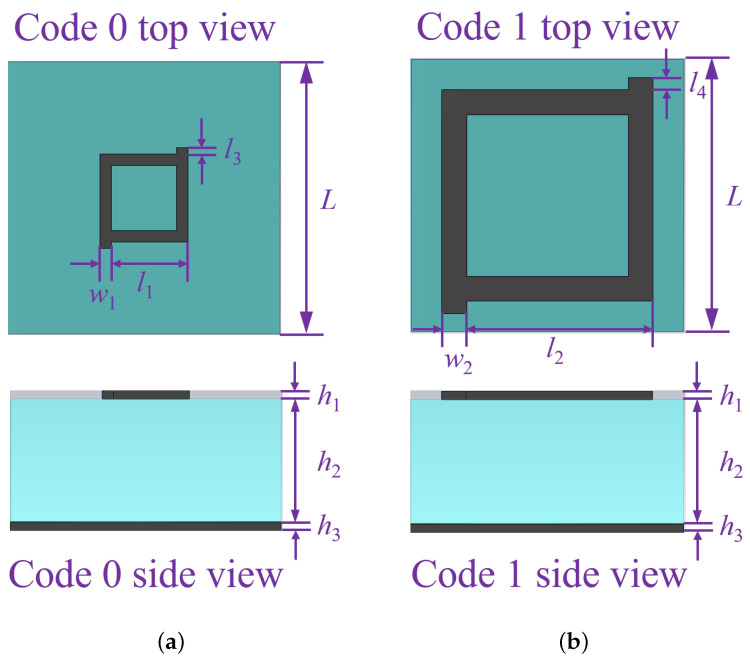
Unit cells structure: dimensions, side view, and top view of unit cells used for this study.

**Figure 4:**
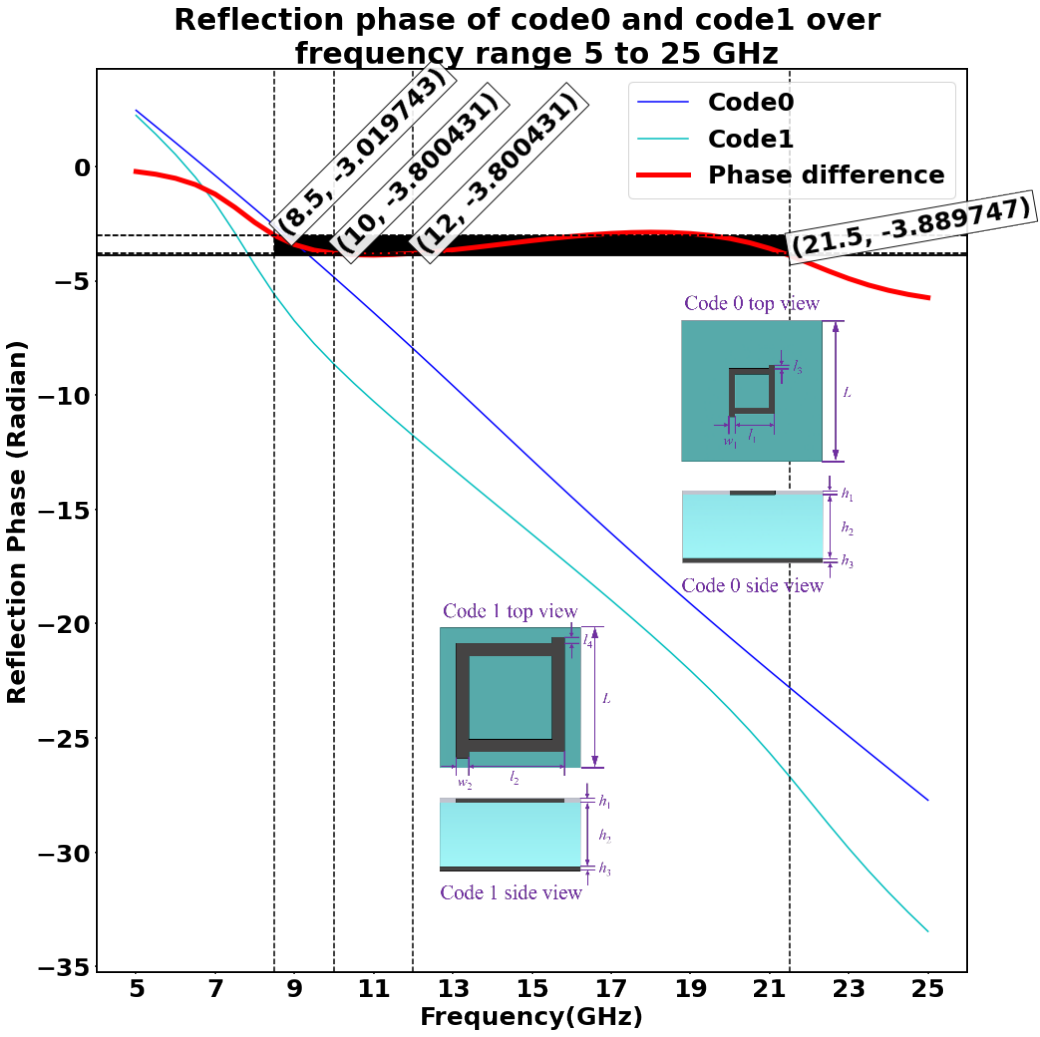
Reflection phase of two unit cells in the frequency range 5–25 GHz. This plot shows the reflection phase of the two unit cells considered over the frequency range 5–25 GHz and their phase difference in the same range of frequency.

The initial dataset used to train the machine learning model is obtained by performing a CST Microwave Studio simulation of a 10 × 10 metasurface array consisting of two unit cells. The overall layout of the metasurface array is as shown in the Figure 2. The two meta atoms, code 0 acting as 0th element and code 1 acting as 1st element, chosen in this study are as shown in Figure 3a,b, respectively. The unit cell consists of two copper layers with thickness $h_{1}$ = $h_{3}$ = 0.02 mm, and a substrate layer is inserted between the two copper layers with thickness $h_{2}$ = 3 mm with permittivity $\epsilon_{r}$ = 2.65 and loss tangent tan(δ) = 0.003. In addition, the side length of each lattice is set to *L* = 6.5 mm, and the parameters of unit 0 and 1 are defined as $l_{1}$ = 2.05 mm, $w_{1}$ = 0.08 mm, $l_{3}$ = 0.15 mm, $l_{2}$ = 4.90 mm, $w_{2}$ = 0.20 mm, and $l_{4}$ = 0.30 mm, respectively. Here, the 0s and 1s are based on wave structure interaction (i.e., 0 and π phase responses, respectively). These elements are chosen because they have a reflection phase difference of 180${}^{\circ }$ for a wide range of frequency from 10 GHz to 12 GHz, as shown in Figure 4; hence, the radar cross-section reduction can be achieved over a wider band of frequencies.

The dataset is composed of 450 data points, with the input features as a sequence of 100 unit cells (10 × 10 array) comprised of a combination of 0 and 1 and the target values as their corresponding radar cross-section from 5 GHz to 25 GHz. The training set consists of 80% of the total number of elements, and the remaining 20% is used as the test set.

#### 2.2.2. Machine Learning Model Design

The work [36] illustrates the mathematical foundation, on which the four pillars of machine learning techniques (regression, dimensionality reduction, density estimation, and classification) are based. In traditional computer programming, a program is coded to produce a desired output. Executing the program with user inputs produces the corresponding outputs. Machine learning techniques are where the computer starts to learn the function that maps the inputs to outputs, to be able to predict the output for unseen input data. In supervised learning, the machine is provided with an input dataset consisting of labelled data (i.e., for every input feature the respective output values will be available). Depending on the type of output value, whether it is continuous numeric value or categories, supervised learning techniques are classified as a regression task or a classification task, respectively. In unsupervised learning, the machine is provided with an unlabelled input dataset.

The machine learning technique selected in this study is the Gaussian process regressor. Different regressor models such as support vector regressor, decision tree regressor, random forest regressor, and gradient boosting regressor were also tested to evaluate the performance, as shown in Table 1. Gaussian process regressor was trained and could make highly accurate predictions. Another reason for choosing Gaussian process regressor is because the active learning strategy needs the machine learning models to make predictions as well as give the uncertainity of predictions.

**Figure 5:**
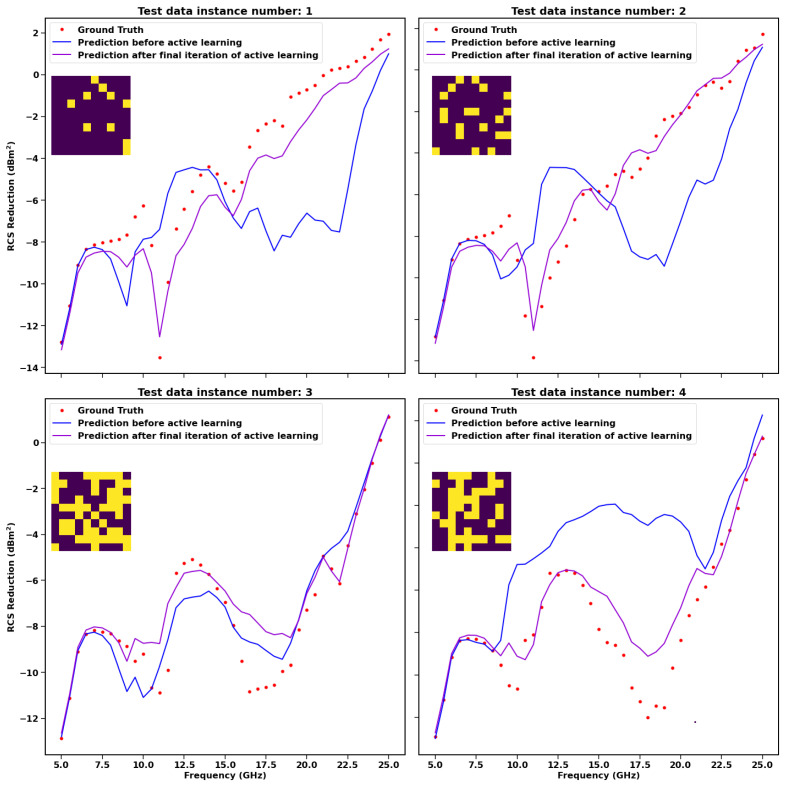
Steps involved in the process of Bayesian optimisation. The figures illustrate four different examples of how the prediction of the model improves after the final iteration of active learning. Ground truth is the RCS reduction obtained for the respective 10 × 10 metasurface array, as shown in the inset with the help of CST simulation.

In Figure 5, the steps involved in the active learning methodology are illustrated. Four different examples of how the machine learning model improves its prediction accuracy after the first and last iteration of active learning have been demonstrated. The blue-coloured line represents the model prediction. The purple line represents how the prediction improves after the final iteration of active learning. The model performs much better after the final iteration by making predictions much closer to ground truth values.

### 2.3. Active Learning Using Surrogate Model

Active learning is tested to significantly lower the computation time taken by the genetic algorithm, especially at a larger population size. An additional method to gain computational time in active learning is by replacing the mode of labelling the data instance obtained after querying the subset of the solution space using an actual CST Microwave Studio simulation, with prediction made by the accurately trained machine learning model. The aforementioned aspect is illustrated in Algorithm 1.
**Algorithm 1** Active learning using surrogate model as the oracle**Input**: Define the parameter space. N×N metasurface array with various combinations of elements 0 and 1.**Perform**: CST simulation to calculate the RCS values over desired frequency range for considered metasurface array.**Collect**: Collect training dataset.**Construct**: Build the appropriate Gaussian process regression model.**while** budget≠0 **do**   **Find**: The metasurface array combination with minimum RCSpredictions in the frequency range in the test set.   **Compute**: Train Gaussian process regressor’s prediction to calculate the RCS value of the metasurface array from the above step.   **Inclusion**: Include the above combination of the metasurface array and its corresponding RCS values in the training dataset.   **Retrain**: Train the model again using the above informative sample and its respective output.   budget←budget−1**end while****Optimal design**: Select the candidate metasurface array contributing to minimum RCS in the desired frequency range.

### 2.4. Genetic Algorithm and Its Set-Up in This Work

Genetic algorithm [37,38] begins with the initialisation step in the first generation. Initialising the population with the required format, consisting of the desired number of chromosomes. Then, for every pair of parents, a crossover operation is performed, resulting in a pair of offspring. Next, mutation of the offspring is performed before merging them with the parents. From the merged set of offspring and the main population, the desired number of the fittest individuals are selected to play the role of parents for the next generation. These steps are repeated either for a required number of generations or until a termination criterion is met. Operating on (potentially large) populations and the repetitive nature of genetic algorithms make it computationally intensive, as well as time consuming, to arrive at the optimal result.

The first step of parent selection can be done in different ways, such as random selection, tournament selection, and roulette wheel selection. In this work, we have used tournament selection as the method. The crossover operation can be performed as single-point crossover, double-point crossover, or uniform crossover. We have used double-point crossover. Then comes the mutation step. The mutation operator helps to periodically and randomly restore the population by introducing new patterns into the chromosomes, enabling search in uncharted areas of the solution space. We have used flip bit mutation. When flip bit mutation is applied to a binary chromosome, one gene is randomly selected, and its value is complemented. The mutation rate we have used is 5%. The optimisation function used is the accurately trained machine learning model, which takes into account mutual coupling between the two unit cells in the metasurface array.

## 3. Results and Discussion

We found that by using active learning, there is significant reduction in computational time for optimisation, especially for a larger metasurface array. In this work, up to a 60 × 60 metasurface array design has been successfully optimised at significantly lower computational time compared to genetic algorithm. The optimal result obtained with the active learning strategy is approximately close to that obtained with the genetic algorithm. A novel strategy of active learning using an accurately trained surrogate model is attempted and validated. The above approach further contributes to lowering computational time.

The population sizes considered in our work is in orders of 10, varying as $10^{3}$, $10^{4}$, $10^{5}$, and $10^{6}$. These population sizes are considered because, for genetic algorithm, the rule of thumb is that the population size should be at least 10 times the dimensionality of the problem under consideration. For a 10 × 10 metasurface array, the number of features is 100. Genetic algorithm needs a minimum population size of 10 × 100, which is $10^{3}$. Thus, the population size begins with $10^{3}$, varying every order of 10 up to $10^{6}$. The better optimal result is obtained when the population size is large, especially for metasurface arrays with larger dimensions.

In the work [39], an optimised coding metasurface was reported. The analytical formula to plot the far field radiation pattern of the digital coding metasurface (with elements 0 and 1 representing reflection phase 0 and π radian, respectively) under normal incidence of plane wave is given in Equation (6) [39]:

(6) $$f\left(\theta ,\phi \right)=f_{e}\left(\theta ,\phi \right)\sum_{m=1}^{N}\sum_{n=1}^{N}\exp\left(-j\left(\varphi \left(m,n\right)+k\,D\,\sin\left(\theta \right)\left(\left(m-\frac{1}{2}\right)\cos\left(\phi \right)+\left(n-\frac{1}{2}\right)\sin\left(\phi \right)\right)\right)\right)$$

where θ and ϕ are the elevation and azimuth angles of an arbitrary direction, respectively; $f_{e}\left(\theta ,\phi \right)$ is the pattern function of a lattice, a general square metasurface that contains N × N equally sized lattices with dimension *D* in which each lattice is occupied by a sub-array of ‘0’ or ‘1’ elements; m is the row number of the array; n is the column number of the array; and *k* is the wave number.

(7) $$Dir\left(\theta ,\phi \right)=\frac{{4\pi |f\left(\theta ,\phi \right)}^{2}|}{\int_{0}^{2\pi }\int_{0}^{\pi /2}\left|f{\left(\theta ,\phi \right)}^{2}\right|\,\sin\left(\theta \right)\,d\theta \,d\phi }$$

Dir(θ,ϕ) is the directivity. The radar cross-section reduction (RCSReduction) is given by the following Equation (8):

(8) $$RCS\;Reduction=\frac{\lambda^{2}}{4\pi \,N^{2}\,D^{2}}Max_{\theta ,\phi }\left(Dir\left(\theta ,\phi \right)\right)$$

The optimisation objective is to reduce the radar cross-section reduction. They have used the analytical formula as given in Equation (8) as the objective function. This method of optimisation uses the analytical formula, which ignores the mutual coupling between the unit cells’ constituents in the metasurface array. Hence, the optimal result does not include mutual coupling between various elements.

In the work [40], the chessboard and their optimally designed coding diffusion metasurface at 15.4 GHz are simulated, and their findings show that for the optimal design, there are eight scatter lobes in the radiation pattern, while the chessboard has only four main lobes. In this work, we tried to compare the result of the optimal design using genetic algorithm and active learning at a population size of $10^{6}$ with chessboard design at 10 GHz. Our result as shown in Table 2 states that there are multiple side lobes for the radiation pattern for the optimal designs obtained from genetic algorithm and active learning, but there are only two main lobes for the chessboard pattern.

**Figure 6:**
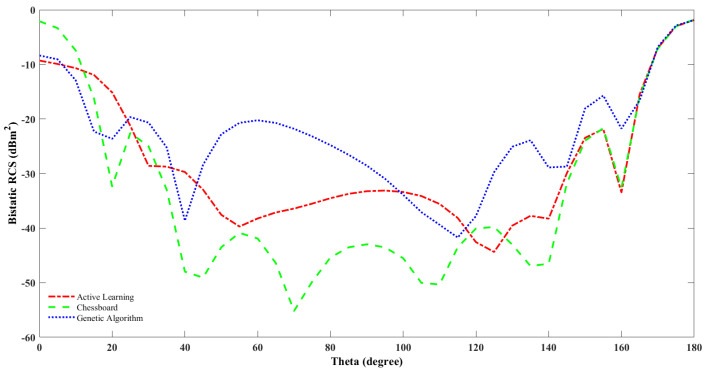
Bistatic RCS of optimal metasurface design using genetic algorithm and active learning, including CST simulation result and chessboard configuration versus theta.

The bandwidth that implies the frequency range where RCS reduction is more is higher for the active learning and genetic algorithm optimally designed metasurface compared to the chessboard metasurface configuration. Monostatic RCS reduction is measured when the field source (e.g., radar beam) and the observation point are at the same location. Bistatic RCS reduction is measured when the field source (e.g., radar beam) and the observation point are at different locations.The bistatic RCS for the chessboard and the optimal design using genetic algorithm and active learning are shown in Figure 6.

In this work, we have shown that active learning plays an important role in significantly reducing the computation time in the optimisation process, by an especially large factor at high population size compared to genetic algorithm. This is illustrated in Table 3. The substitution of one of the steps in active learning—that is, performing CST Microwave Studio simulation for the selected data instance after querying the subset of the solution space, with an accurately trained surrogate model to make an equivalent prediction—contributes to saving computational time. The optimisation procedure is performed after taking into consideration the mutual coupling between unit cells.

The work can be extended by considering more than two different unit cells. This is because the digital bits represent the wave structure interaction, which offers multiple degrees of freedom [41]. For example, instead of elements ‘0’ and ‘1’, there can be more elements: ‘0’, ‘1’, ‘2’, and ‘3’. An equivalent initial dataset with a combination of elements and corresponding radar cross-section over the desired range of frequency has to be prepared. Different geometry unit cells can also be considered for study. We have extended the method up to 40 × 40 and 60 × 60 metasurface arrays. In order to perform optimisation of 40 × 40 and 60 × 60 metasurface arrays, we have created a dataset that is available with similar dimensions, simulated using CST Microwave Studio to train the machine learning model that will be used in the active learning strategy. Research can be conducted on figuring out ways to extend it further for larger scales of metasurface array.

## 4. Fabrication and Measurement

**Figure 7:**
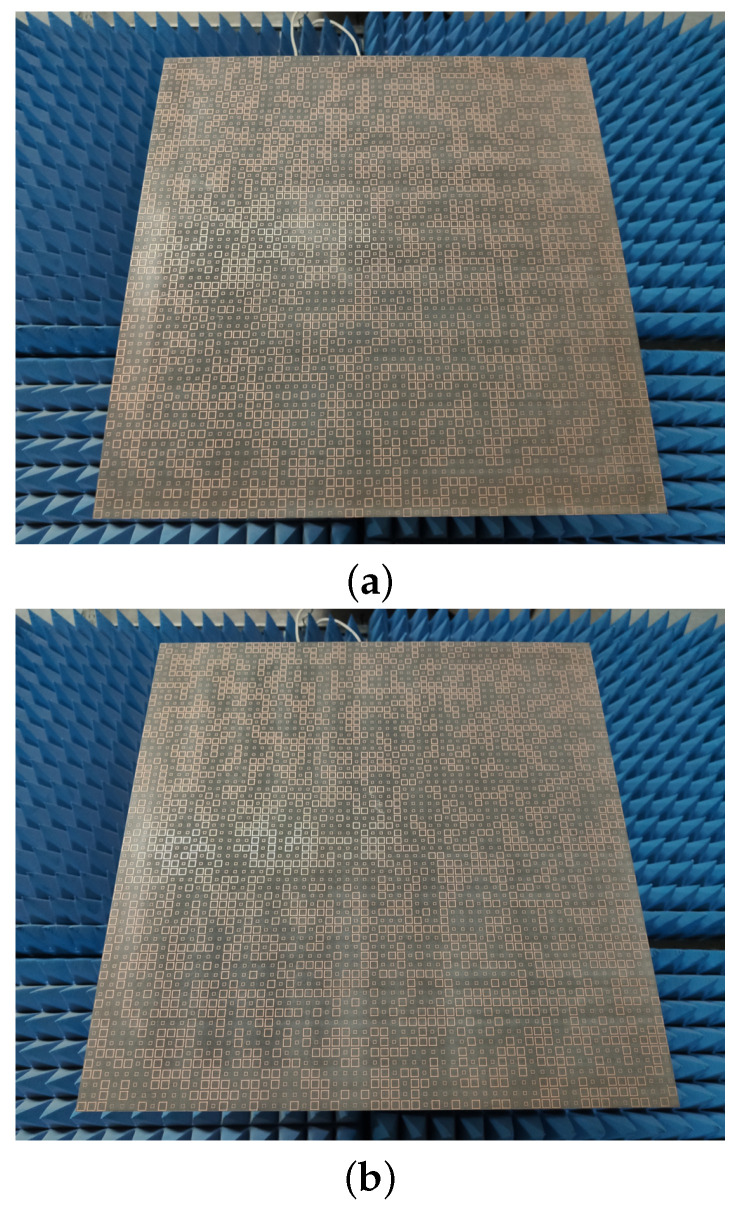
Fabricated samples of optimal design: (**a**) using genetic algorithm; (**b**) using active learning.

To verify the optimisation results, a prototype of the proposed metasurface for a 60 × 60 metasurface array is illustrated in Figure 7 and has been experimentally studied. The diffuse metasurface sample was fabricated using an optical lithographic process on a 3 mm thick F4B substrate with $\epsilon_{r}$ = 2.65 and loss tangent tan(δ) = 0.003.

In this experiment, two broadband horn antennas operating from 10 GHz to 20 GHz connected to a vector network analyser (VNA) are used as transmitter and receiver antennas, respectively. The fabricated sample is kept in the centre of the arch. The transmitter antenna is fixed to the top of the sample to generate a quasi-plane wave, and the receiver antenna can move along the arch to obtain reflective signals of the sample on the azimuth plane. The setup needs time gating, which only allows a narrow measurement bandwidth. Hence, measurement is repeated multiple times to cover the required frequency range.

**Figure 8:**
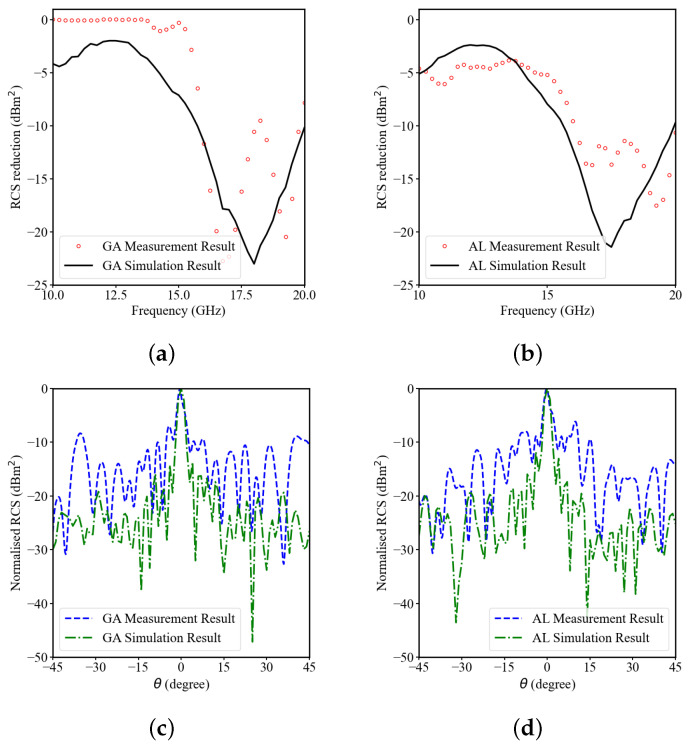
Optimal design fabricated sample simulation and measurement results: (**a**) using genetic algorithm: RCS reduction versus frequency; (**b**) using active learning: RCS reduction versus frequency; (**c**) using genetic algorithm: normalised RCS versus angle; (**d**) using active learning: normalised RCS versus angle.

Figure 8a,b shows the measured and simulated result of RCS reduction versus frequency curves. The active learning methodology does not contribute to any of the mismatch between the measurement and simulation results. The difference between the measurement and simulation results is attributed to the mutual coupling occurring between the transmitter and receiver antenna. In future work, in order to minimise the difference, the mutual coupling between transmitter and receiver antenna needs to be incorporated while performing the simulation. From the results, the bandwidth of the RCS reduction greater than 10 dB is 16–20 GHz, which is in agreement with the simulated result. Figure 8c,d illustrates the normalised RCS versus angle, measured at frequency 14 GHz. Neglecting fabrication and measurement errors, the measured results verify the low scattering of this novel design.

## 5. Conclusions

**Figure 9:**
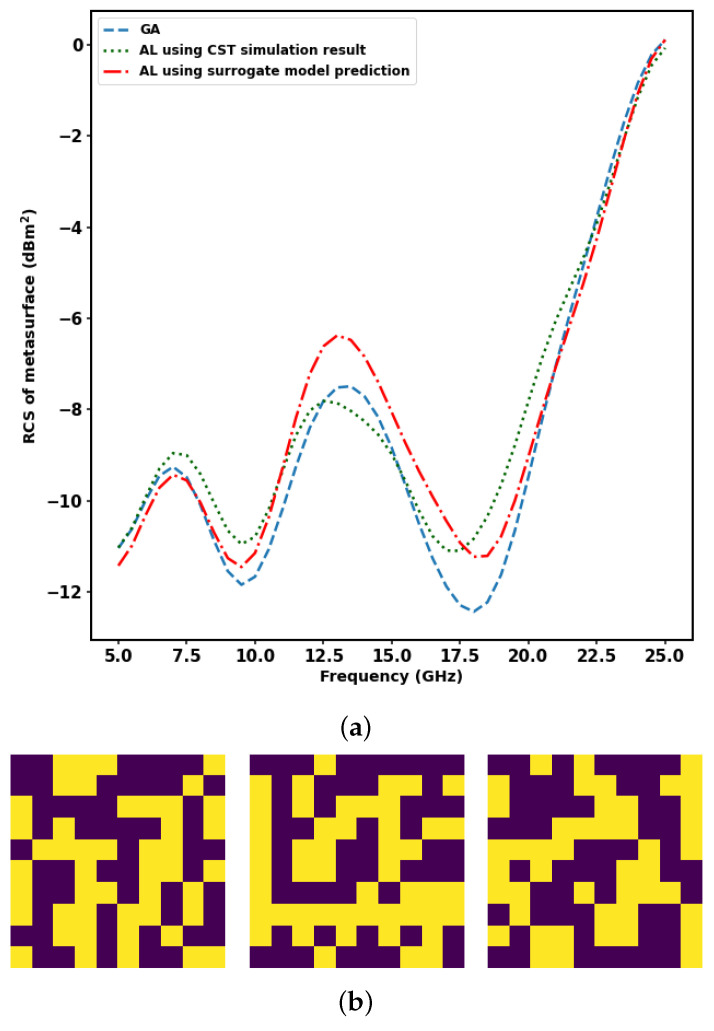
Optimal RCS versus frequency and corresponding optimal design using methods of optimisation considered in this study at a population size of $10^{6}$. (**a**) Comparison of radar cross-section versus frequency for the optimal design obtained from genetic algorithm and active learning with and without the surrogate model at a population size of $10^{6}$. (**b**) Optimal design obtained from genetic algorithm and active learning with and without the surrogate model, respectively, at a population size of $10^{6}$.

Active learning plays an important role in lowering the computational time required to perform metasurface array design with the optimisation objective of minimizing the radar cross-section by giving optimal results approximately similar to the genetic algorithm. As an example, a 10 × 10 metasurface array is considered. Genetic algorithm and active learning using a surrogate model and active learning using CST Microwave Studio simulation obtain similar results. Better optimal results are obtained when the population size is larger, as shown in Figure 9a, and at a huge population size, active learning significantly overtakes genetic algorithm in computational time. The optimal design using genetic algorithm, active learning using a surrogate model, and active learning using CST Microwave Studio simulation are as shown in Figure 9b. The computational time gained when using active learning compared to genetic algorithm at a large population size of $10^{6}$ is shown in Table 3.

In active learning, when the predictions made by an accurately trained surrogate machine learning model are used in the place of CST Microwave Studio simulation results, there is further scope for lowering the magnitude of computational time. Using surrogate model predictions, the active learning method of metasurface array design can be extended to larger scales of metasurface array design. In this work, we have verified this at the scale of 60 × 60. The mutual coupling between unit cells is also considered while designing the optimal metasurface array.

## Figures and Tables

**Table 1:** Comparison of performance metrics of different regressor models for 10 × 10 metasurface array.

Performance Metrics	Regressors				
	Support Vector Regressor	Decision Tree	Random Forest	Gradient Boosting	Gaussian Process
Mean Absolute Error	0.6322	1.3728	0.8159	0.7813	0.5871
Mean Squared Error	0.9216	3.8664	1.3917	1.3830	0.8190
R${}^{2}$ Score *	0.9111	0.5321	0.8261	0.8269	0.9216

* In regression, the R${}^{2}$ score, which is the coefficient of determination, is a statistical measure of how well the regression predictions approximate the real data points.

**Table 2:** Comparison of active learning using CST Microwave Studio simulation, genetic algorithm and chess board configuration characteristics.

Technique	Bandwidth	Scattering Field Lobes	Monostatic RCS Reduction (dBm${}^{2}$)	Bistatic RCS Reduction at j = 0${}^{\circ }$ & 150${}^{\circ }$ (dBm${}^{2}$)	Operating Bandwidth
Active learning designed metasurface	95%	Multiple lobes	−11.2100	−11.9500 & −48.0300	5–20 GHz
Genetic algorithm designed metasurface	92%	Multiple lobes	−12.0800	−9.8340 & −37.5600	5–20 GHz
Chessboard configuration	26.67%	Two Lobes	−5.5138	−5.5140 & −43.3500	5–9 GHz

**Table 3:** Comparison of active learning and genetic algorithm.

Population Size	Computational Time (Minutes)	
	Active Learning	Genetic Algorithm
$10^{3}$	23.29	0.09
$10^{4}$	30.78	1.21
$10^{5}$	32.23	89
$10^{6}$	65	13260

## Data Availability

All relevant data are available from the corresponding author on request.

## References

[1] Liu Y., Zhang X. (2011). Metamaterials: A new frontier of science and technology. Chem. Soc. Rev..

[2] Sun S., He Q., Hao J., Xiao S., Zhou L. (2019). Electromagnetic metasurfaces: Physics and applications. Adv. Opt. Photonics.

[3] Huang S., Xie Z., Chen W., Lei J., Wang F., Liu K., Li L. (2018). Metasurface with multi-sized structure for multi-band coherent perfect absorption. Opt. Express.

[4] Li L., Jun Cui T., Ji W., Liu S., Ding J., Wan X., Bo Li Y., Jiang M., Qiu C.W., Zhang S. (2017). Electromagnetic reprogrammable coding-metasurface holograms. Nat. Commun..

[5] Hsiao H.H., Chu C.H., Tsai D.P. (2017). Fundamentals and Applications of Metasurfaces. Small Methods.

[6] Chen J., Cheng Q., Zhao J., Dong D.S., Cui T.J. (2014). Reduction of Radar Cross Section based on a metasurface. Prog. Electromagn. Res..

[7] Al-Nuaimi M.K., Whittow W.G. (2022). Design of QR-Coded Metasurfaces for RCS Reduction at mmWave. IEEE Access.

[8] Zhou Y., Cao X.Y., Gao J., Li S., Liu X. (2017). RCS reduction for grazing incidence based on coding metasurface. Electron. Lett..

[9] Jafar-Zanjani S., Inampudi S., Mosallaei H. (2018). Adaptive Genetic Algorithm for Optical Metasurfaces Design. Sci. Rep..

[10] Elsawy M.M., Lanteri S., Duvigneau R., Fan J.A., Genevet P. (2020). Numerical Optimization Methods for Metasurfaces. Laser Photonics Rev..

[11] Christiansen R.E., Sigmund O. (2020). A tutorial for inverse design in photonics by topology optimization. arXiv.

[12] Jensen J.S., Sigmund O. (2011). Topology Optimization for Nano-Photonics. Laser Photonics Rev..

[13] Zhu X., Shao W., Li J.L., Dong Y.L. (2012). Design and Optimization of Low Rcs Patch Antennas Based on a Genetic Algorithm. Prog. Electromagn. Res..

[14] Robinson J., Rahmat-Samii Y. (2004). Particle swarm optimization in electromagnetics. IEEE Trans. Antennas Propag..

[15] Bossard J.A., Lin L., Yun S., Liu L., Werner D.H., Mayer T.S. (2014). Near-Ideal Optical Metamaterial Bandwidth. ACS Nano.

[16] Wu W., Dunlop J.B., Collocott S.J. (2003). Design optimization of switched reluctance motor by electromagnetic and thermal finite element analysis. Proceedings of the Intermag 2003—Program of the 2003 IEEE International Magnetics Conference.

[17] Jiang J., Fan J.A. (2019). Global optimization of dielectric metasurfaces using a physics-driven neural network. Nano Lett..

[18] Kiarashinejad Y., Abdollahramezani S., Zandehshahvar M., Hemmatyar O., Adibi A. (2019). Deep Learning Reveals Underlying Physics of Light-matter Interactions in Nanophotonic Devices. Adv. Theory Simul..

[19] Nadell C.C., Huang B., Malof J.M., Padilla W.J. (2019). Deep learning for accelerated all-dielectric metasurface design. Optics Express.

[20] An S., Zheng B., Shalaginov M.Y., Tang H., Li H., Zhou L., Ding J., Agarwal A.M., Rivero-Baleine C., Kang M. (2020). Deep learning modeling approach for metasurfaces with high degrees of freedom. Opt. Express.

[21] da Silva Ferreira A., da Silva Santos C.H., Gonçalves M.S., Hernández Figueroa H.E. (2018). Towards an integrated evolutionary strategy and artificial neural network computational tool for designing photonic coupler devices. Appl. Soft Comput. J..

[22] Turduev M., Bor E., Latifoglu C., Giden I.H., Sinan Hanay Y., Kurt H. (2018). Ultracompact Photonic Structure Design for Strong Light Confinement and Coupling Into Nanowaveguide. J. Light. Technol..

[23] Xu L., Rahmani M., Ma Y., Smirnova D.A., Kamali K.Z., Deng F., Chiang Y.K., Huang L., Zhang H., Gould S. (2019). Enhanced light-matter interactions in dielectric nanostructures via machine learning approach. arXiv.

[24] Costa F., Monorchio A., Manara G. (2016). Wideband Scattering Diffusion by using Diffraction of Periodic Surfaces and Optimized Unit Cell Geometries. Sci. Rep..

[25] Han T., Cao X.Y., Gao J., Zhao Y.L., Zhao Y. (2017). A coding metasurface with properties of absorption and diffusion for RCS reduction. Prog. Electromagn. Res. C.

[26] Jidi L., Cao X., Tang Y., Wang S., Zhao Y., Zhu X. (2018). A new coding metasurface for wideband RCS reduction. Radioengineering.

[27] Lookman T., Balachandran P.V., Xue D., Yuan R. (2019). Active learning in materials science with emphasis on adaptive sampling using uncertainties for targeted design. NPJ Comput. Mater..

[28] Jones D.R., Schonlau M., Welch W.J. (1998). Efficient Global Optimization of Expensive Black-Box Functions. J. Glob. Optim..

[29] Kusne A.G., Yu H., Wu C., Zhang H., Hattrick-Simpers J., DeCost B., Sarker S., Oses C., Toher C., Curtarolo S. (2020). On-the-fly closed-loop materials discovery via Bayesian active learning. Nat. Commun..

[30] Schmidt J., Marques M.R., Botti S., Marques M.A. (2019). Recent advances and applications of machine learning in solid-state materials science. NPJ Comput. Mater..

[31] Budd S., Robinson E.C., Kainz B. (2021). A survey on active learning and human-in-the-loop deep learning for medical image analysis. Med. Image Anal..

[32] De Angeli K., Gao S., Alawad M., Yoon H.J., Schaefferkoetter N., Wu X.C., Durbin E.B., Doherty J., Stroup A., Coyle L. (2021). Deep active learning for classifying cancer pathology reports. BMC Bioinform..

[33] El-Hasnony I.M., Elzeki O.M., Alshehri A., Salem H. (2022). Multi-Label Active Learning-Based Machine Learning Model for Heart Disease Prediction. Sensors.

[34] Zhang J., Gong W., Yue X., Shi M., Chen L. (2022). Efficient reliability analysis using prediction-oriented active sparse polynomial chaos expansion. Reliab. Eng. Syst. Saf..

[35] Rubens N., Elahi M., Sugiyama M., Kaplan D., Ricci F., Rokach L., Shapira B. (2015). Active Learning in Recommender Systems. Recommender Systems Handbook.

[36] Deisenroth M.P., Faisal A.A., Ong C.S. (2020). Mathematics for Machine Learning.

[37] Kramer O. (2017). Studies in Computational Intelligence 679 Genetic Algorithm Essentials.

[38] Katoch S., Chauhan S.S., Kumar V. (2020). A review on genetic algorithm: Past, present, and future. Multimed. Tools Appl..

[39] Cui T.J., Qi M.Q., Wan X., Zhao J., Cheng Q. (2014). Coding metamaterials, digital metamaterials and programmable metamaterials. Light.

[40] Ali L., Li Q., Khan T.A., Yi J., Chen X. (2019). Wideband RCS reduction using coding diffusion metasurface. Materials.

[41] Bai G.D., Cui T.J. (2020). Representing Quantum Information with Digital Coding Metasurfaces. Adv. Sci..

